# Haematological spectrum and genotype-phenotype correlations in nine unrelated families with *RUNX1* mutations from the French network on inherited platelet disorders

**DOI:** 10.1186/s13023-016-0432-0

**Published:** 2016-04-26

**Authors:** Veronique Latger-Cannard, Christophe Philippe, Alexandre Bouquet, Veronique Baccini, Marie-Christine Alessi, Annick Ankri, Anne Bauters, Sophie Bayart, Pascale Cornillet-Lefebvre, Sylvie Daliphard, Marie-Joelle Mozziconacci, Aline Renneville, Paola Ballerini, Guy Leverger, Hagay Sobol, Philippe Jonveaux, Claude Preudhomme, Paquita Nurden, Thomas Lecompte, Remi Favier

**Affiliations:** Service d’Hématologie Biologique, Centre Hospitalier Universitaire de Nancy, Nancy, France; Centre de Compétence Nord-Est des Pathologies Plaquettaires (CCPP), Nancy, France; Laboratoire de Génétique, Centre Hospitalier Universitaire de Nancy, Nancy, France; Service d’Hématologie Biologique, Centre de Biologie Pathologie, Centre Hospitalier Régional Universitaire de Lille, Lille, France; Laboratoire d’Hématologie, Hôpital La Timone, Marseille, France; Assistance Publique-Hôpitaux de Paris, Laboratoire d’Hématologie, La Pitié Salpetrière, Paris, France; Centre Régional de Traitement des Hémophiles, Centre Hospitalier Universitaire de Rennes, Rennes, France; Laboratoire d’Hématologie, Centre Hospitalier Universitaire Robert Debré, Reims, France; Département de Biopathologie, Institut Paoli-Calmettes, Centre de Recherche en Cancérologie de Marseille, Marseille, France; Service d’Hématologie, Hôpitaux Universitaires de Genève, Geneva, Switzerland; Faculté de Médecine, Université de Genève, Geneva, Switzerland; Assistance Publique-Hôpitaux de Paris, Département d’Hématologie, Hôpital Armand Trousseau, Paris, France; Inserm U1170, Villejuif, France; Centre de Référence des Pathologies Plaquettaires (CRPP), Hôpital La Timone, Marseille, France; Service d’Hématologie Biologique, Hôpital d’enfants Armand Trousseau, 26 Avenue du Dr Netter, 75012 Paris, France

**Keywords:** Thrombocytopenia, Familial platelet disorder with predisposition to acute myeloid leukaemia, RUNX1, Leukaemia, δ-granule release defect

## Abstract

**Background:**

Less than 50 patients with FPD/AML (OMIM 601309) have been reported as of today and there may an underestimation. The purpose of this study was to describe the natural history, the haematological features and the genotype-phenotype correlations of this entity in order to, first, screen it better and earlier, before leukaemia occurrence and secondly to optimize appropriate monitoring and treatment, in particular when familial stem cell transplantation is considered.

**Methods:**

We have investigated 41 carriers of *RUNX1* alteration belonging to nine unrelated French families with FPD/AML and two syndromic patients, registered in the French network on rare platelet disorders from 2005 to 2015.

**Results:**

Five missense, one non-sense, three frameshift mutations and two large deletions involving several genes including *RUNX1* were evidenced. The history of familial leukaemia was suggestive of FPD/AML in seven pedigrees, whereas an autosomal dominant pattern of lifelong thrombocytopenia was the clinical presentation of two. Additional syndromic features characterized two large sporadic deletions. Bleeding tendency was mild and thrombocytopenia moderate (>50 x10^9^/L), with normal platelet volume. A functional platelet defect consistent with a δ-granule release defect was found in ten patients regardless of the type of *RUNX1* alteration. The incidence of haematological malignancies was higher when the mutated *RUNX1* allele was likely to cause a dominant negative effect (19/34) in comparison with loss of function alleles (3/9). A normal platelet count does not rule out the diagnosis of FPD/AML, since the platelet count was found normal for three mutated subjects, a feature that has a direct impact in the search for a related donor in case of allogeneic haematopoietic stem cell transplantation.

**Conclusions:**

Platelet dysfunction suggestive of defective δ-granule release could be of values for the diagnosis of FPD/AML particularly when the clinical presentation is an autosomal dominant thrombocytopenia with normal platelet size in the absence of familial malignancies. The genotype-phenotype correlations might be helpful in genetic counselling and appropriate optimal therapeutic management.

## Background

Four syndromes due to inheritance of a single abnormal copy of a gene coding for a transcription factor that is critical for haematopoiesis have been identified as predisposition syndromes for familial myelodysplastic syndrome (MDS) or acute leukaemia (AL). The involved genes are *RUNX1* (familial platelet disorder with propensity to develop acute myeloid leukaemia, FPD/AML, OMIM 601399) [1], *CEBPA* [2] and more recently *GATA-2* [3] and *ETV6* [4]. The clinical usefulness of identifying these syndromes, which may be more common than previously thought, is obvious. They raise questions about their clinical detection and management. Heterozygous germline nonsense or missense mutations in *RUNX1* were identified as the causative abnormality in FPD/AML, the first described of the four [1]. Less than 50 mutation-positive pedigrees have been reported in the literature [5]. Whole or partial hemizygous *RUNX1* deletions can also occur [6]. Recently, some intragenic deletions and duplications undetectable by sequencing were reported [7]. Platelet abnormalities participate to the definition of FPD/AML [8, 9]. Time elapsed between the detection of thrombocytopenia and the diagnosis of FPD/AML can amount to several years: thus FPD/AML diagnosis remains a challenge. This is of utmost importance when an intra-familiar donor is considered for hematopoietic stem cell transplantation (HSCT) [10].

By retrospectively collecting and analysing the data of 41 affected subjects from nine unrelated French families and two syndromic patients, we mainly aimed at improving the characterization of the clinical and laboratory phenotype of FPD/AML in order to identify some clues, readily obtained in clinical practice, for appropriate recognition of this syndrome. This group of patients with germline *RUNX1* mutations or deletions is the largest collected up to now.

## Methods

### Organization of the French registry and data monitoring

All patients included in this study were registered in the French network on rare platelet disorders: ‘Centre de Reference des Pathologies Plaquettaires’, founded in 2004 and based on prospective enrolment. All types of inherited platelet disorders have been included. The registry was started in 2008. Five Reference Centres and two Competence Centres of inherited platelet disorders have participated in the registry. Clinical and laboratory data monitoring was based on the retrospective review of medical records by each physician in charge of the patient or by a clinical research associate. The patient or his- her legal guardians provided written informed consent before being included in the registry. Informed consent were obtained before peripheral blood and bone marrow sampling from all the studied members of the families or the parents of the children in accordance with the Declaration of Helsinki. Some patients were previously and partially reported but without detailed information neither on platelet and megakaryocyte phenotypes nor on clinical follow up: pedigrees B and F [11]; pedigrees C and I [12]; pedigree H and patient J [13].

The collection of these data reflects the collaboration of French centres in the frame of a network on platelet disorders.

### Clinical investigation

The FPD/AML manifestations mainly consist in haematological features which were collected in this study. Bleeding symptoms were evaluated according to the International Society of Thrombosis and Haemostasis (ISTH)- bleeding assessment tool (BAT) [14]. The age and the mode of discovery of the thrombocytopenia, the age at which leukaemia (AL) arose and its type (according to OMS 2008 classification), the treatment by familial or unrelated donor allogeneic HSCT and the lapse of time between thrombocytopenia detection and FPD/AML diagnosis were collected. Others features were reported for syndromic forms.

### Platelet and bone marrow investigations

Platelet count and mean platelet volume (MPV) were measured with ethyldiaminetetraacetic acid (EDTA) anticoagulated peripheral blood in each centre with its haematological analyser. Platelet volume and size were analysed according to the French Platelet Reference Centre recommendations, taking into account the differences between cell counters [15]. Platelet functional investigations were performed for eight pedigrees (A to I except B, 20 patients) and patient J avoiding prior intake of aspirin and non-steroidal anti-inflammatory agents. Peripheral blood was anticoagulated with 3.8 % sodium citrate. Platelet aggregation was evaluated by light transmission aggregometry (LTA) with platelet rich plasma (PRP) in response to several agonists at different concentrations depending on the laboratory: arachidonic acid (AA) (1.36 mM), adenosine diphosphate (ADP) (2; 5; 10 μM), collagen (2; 10 μg/mL), epinephrine (1.2; 2.4; 5.0; 10.0; 50.0 μM) and agglutination in the presence of ristocetin (0.5; 0.8; 1.5 mg/mL). Dense (δ)-granules and release of their content were investigated by various means: ATP release during platelet aggregation in a lumiaggregometer (Chrono-Log) with the luciferin-luciferase reagent [16]; flow cytometry assay using mepacrine (a compound selectively taken up into δ-granules) as previously described [17] in combination with CD63 (granulophysin, a membrane marker of δ-granules) expression after stimulation [18]; serotonin content; or electron microscopy (EM). Flow cytometric quantitation of glycoproteins GPIbα (CD42b), GPIIb (CD41a) and GMP140 (CD62P) on the platelet surface with or without activation with thrombin receptor activation (TRAP) was performed with the PLT Gp/Receptors kit (Biocytex, Marseille, France).

Bone marrow aspirate smears, when performed, were stained with May-Grünwald-Giemsa.

### *RUNX1* analysis

*RUNX1* analyses were performed in haematological and genetic laboratories (Lille, Marseille, Nancy, Paris-Trousseau).

#### *Mutation* s*creening*

Total genomic DNA was extracted from peripheral blood using the Nucleon™ BACC genomic DNA extraction kit (GE Healthcare). The coding region between exons 1 to 8 of *RUNX1* was amplified by PCR and directly sequenced as described [11]. Sequence variants were numbered starting from the first base of the ATG codon, numbering based on reference sequence NM_1754.4 (this splicing variant represents the c isoform). Variant calling was performed with the Alamut 2.3.1 software (Interactive Biosoftware), in accordance with the Human Genome Variation Society nomenclature.

#### Comparative genomic hybridization (CGH) array

Deletions in the 21q22 region were identified by CGH-array analysis with the Agilent kit 244A (Agilent Technologies, Santa Clara, CA) or the GenoSensor array (Vysis Inc, Abbot Laboratories SA, Downers Grove, IL, USA) in pedigrees G and H respectively. CGH-array analysis was performed as previously described [13]. The array was analysed with an Agilent scanner and the Feature Extraction software (v10.7.3.1). A graphical overview was obtained using CGH analytics software (v4.0.76).

#### Real-time quantitative PCR

Validation of copy number variations (CNVs) identified with CGH-array was performed by real-time quantitative PCR on genomic DNA, using the ABI PRISM 7500 Sequence Detection System (Applied Biosystems, Foster City, CA) [13]. For each new CNV, we tested three primer sets located in the chromosomal region of interest to establish the *de novo* inherited feature of the chromosomal imbalance.

### Literature review

In order to identify all publications related to FPD/AML syndrome, we screened PubMed, with the key words: thrombocytopenia, leukaemia and *RUNX1.* We then checked the bibliography of each article in order to identify additional references and to avoid duplicates.

## Results

### Clinical and haematological data

The French FPD/AML cohort consists of nine pedigrees (A to I) and two syndromic patients (J and K) with germline *RUNX1* alterations identified in eight hospitals (La Timone and Paoli-Calmettes in Marseille; Nancy; La Pitié-Salpetrière, Saint-Louis and Trousseau in Paris; Reims, Rennes).

**Fig. 1:**
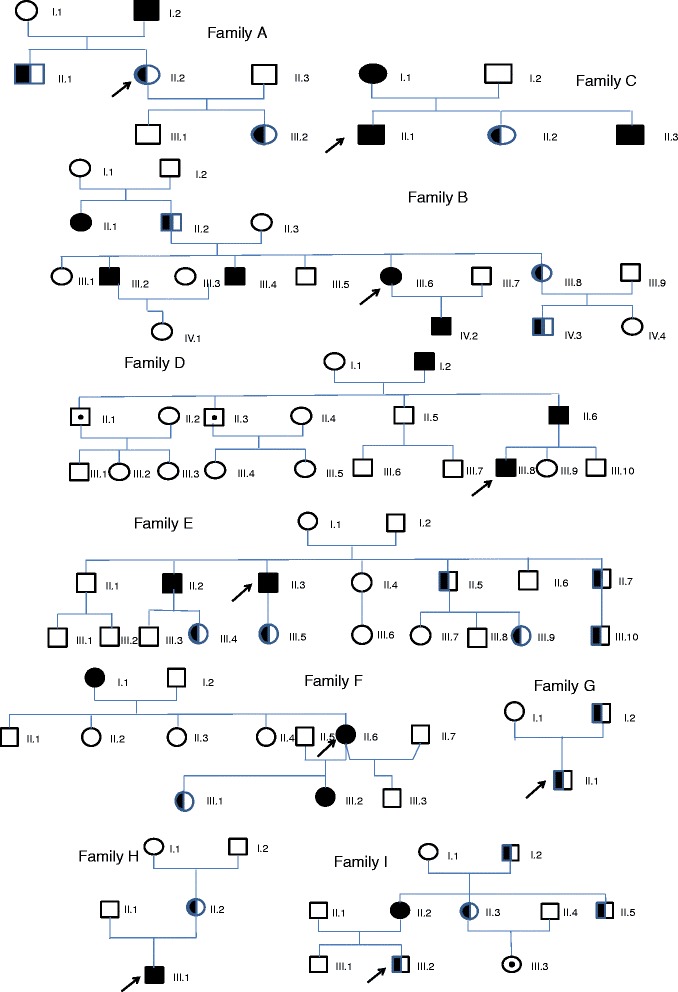
Description of the nine pedigrees with FPD/AML.  : Healthy relatives.  : Mutated patient without thrombocytopenia.  : Mutated patient with thrombocytopenia.  : Patient diagnosed with malignancy. In each family a black arrow designs the proband

Only family pedigrees are shown in Fig. 1 as for patients J and K the genetic defect consists of a *de novo*, sporadic, large deletion. The natural history and the main haematological features are reported below and are summarized in Table 1. The first family member mentioned below is the proband of each pedigree (Table 1). The age at thrombocytopenia diagnosis varied from two to 67 years. When assessed, the ISTH-BAT was found to be between 0 and 4 in all patients but one (E/III:5), who presented with menorrhagia and had a bleeding score of 8.

**Table 1:** Summary of the haematological features of the cohort of 43 patients with FPD/AML syndrome associated with *RUNX1* mutations

Pedigree/patient	Sex	Bleeding score	Age at thrombocytopenia diagnosis	Platelet investigations					AL diagnosis/Age at diagnostic	FPD/AML diagnosis delay	Vital status
				Thrombocytopenia Platelet count (x10^9^/L)	LTA	ATP secretion	Platelet dense content	Bone marrow megacaryopoïesis			
A/I:2	M	NP	ND	Mild thrombocytopenia	NP	NP	NP	NP	AML-M0 and UC-AML 33 and 39	39	Death
A/II:1	M	1	?	70	NP	NP	NP	NP	No hematological malignancy	-	Alive
A/II:2 proband	F	1	?	70	Normal pattern	NP	NP	NP	No hematological malignancy	-	Alive
A/III:2	F	1	?	82	NP	NP	NP	NP	NP	-	Alive
B/II:1	F	NP	ND	Mild thrombocytopenia	NP	NP	NP	NP	UC-AML	-	Death
B/II:2	M		ND	Mild thrombocytopenia	NP	NP	NP	NP	No hematological malignancy	-	Death
B/III:2	M	NP	25	Mild thrombocytopenia	NP	NP	NP	NP	AML-M5 25	<1 year	Death
B/III:4	M	NP	25	Mild thrombocytopenia	NP	NP	NP	NP	AML-M5 25	<1 year	Death
B/III:6 proband	F	NP	41	Mild thrombocytopenia	NP	NP	NP	NP	AML-M0 42	<1 year	Death
B/III:8	F	NP	ND	Mild thrombocytopenia	NP	NP	NP	NP	No hematological malignancy	-	Alive
B/IV:2	M	NP	6	Mild thrombocytopenia	NP	NP	NP	NP	AML-M4 6	<1 year	Death
B/IV:3	M	NP	ND	Mild thrombocytopenia	NP	NP	NP	NP	No hematological malignancy	-	Alive
C/I:1	F	NP	46	84	Normal pattern	NP	Mepacrine and CD63 decreased	Dysmegakaryopoiesis	secondary AML to MDS 56	10 years	Death
C/II:1 proband	M	1	25	119	Normal pattern	NP	Mepacrine decreaed	Dysmegakaryopoiesis	T-ALL and AML-M1 41 and 43	16 years	Death
C/II:2	F	1	18	134	NP	NP	Mepacrine decreaed	Dysmegakaryopoiesis	No hematological malignancy	.-	Alive
C/II:3	M	NP	ND	50	NP	NP	NP	ND	AML-M1 15	ND	Death
D/I:2	M	NP	ND	ND	NP	NP	NP	NP	UC- AML 60	ND	Death
D/II:1	M	0	NA	Normal platelet count	NP	NP	NP	NP	No hematological malignancy	-	Alive
D/II.:3	M	0	NA	Normal platelet count	NP	NP	NP	NP	No hematological malignancy	-	Alive
D/II:6	M	0	47	90–120	δ-SPD pattern	NP	NP	NP	AML-M4 51	4 years	Alive in CR
D/III:8 proband	M	2	8	90–120	δ-SPD pattern	NP	NP	NP	T-ALL 28	20 years	Alive in CR
E/II.2	M	NP	‘Since childhood’	Mild thrombocytopenia	NP	NP	NP	NP	AML	ND	Death
E/II:3 proband	M	NP	‘Since childhood’	122	δ-SPD pattern	NP	Mepacrine and CD63 decreased	Dysmegakaryopoiesis	MDS	-	Death (buccal neoplasia)
E/II/5	M	4	Since childhood	125	NP	NP	NP	NP	No haematological malignncy	ND	Alive
E/II:7	M	3	‘Since childhood’	112	δ-SPD pattern	Decreased	Mepacrine and CD63 decreased	NP	No hematological malignancy	-	Alive
E/III:4	F	2	‘Since childhood’	120	NP	NP	NP	NP	No hematological malignancy	-	Alive
E/III:5	F	8	‘Since childhood’	145	δ-SPD pattern	Decreased	Mepacrine and CD63 decreased	NP	No hematological malignancy	-	Alive
E/III:9	F	3	‘Since childhood’	118	δ-SPD pattern	Decreased	Mepacrine and CD63 decreased	NP	No hematological malignancy	-	Alive
E/III:10	M	3	‘Since the birth’	126	δ-SPD pattern	Decreased	Mepacrine and CD63 decreased	NP	No hematological malignancy	-	Alive
F/I:1	F	NP	‘Since childhood’	Mild thrombocytopenia	NP	NP	NP	NP	CLL	-	Alive
F/II:6 proband	F	3	‘Since childhood’	90–120	NP	NP	NP	NP	AML-M5 55	55	Alive
F/III:1	F	3	‘Since childhood’	120–150	δ-SPD pattern	NP	NP	NP	No hematological malignancy	-	Alive
F/III:2	F	3	‘Since childhood’	Mild thrombocytopenia	NP	NP	NP	NP	T-ALL and AML-M0 18 and 23	18	Death
G/I:2	M	0	‘Since childhood’	70	δ-SPD pattern	NP	Mepacrine and serotonin decreased with normal CD63 expression	NP	No hematological malignancy	-	Alive
G/II:1 proband	M	0	12	79–88	δ-SPD pattern	NP	Normal	NP	No hematological malignancy	-	Alive
H/II:2	F	1	30	120–150	δ-SPD pattern	Decreased	Mepacrine and CD63 decreased	NP	No hematological malignancy	-	Alive
H/III:1 proband	M	2	2	60–90	δ-SPD pattern	Decreased	Mepacrine and CD63 decreased	Dysmegakaryopoiesis	AML-M2 6	4	Alive
I/I:2	M	0	67	120–180	NP	NP	NP	NP	No hematological malignancy	-	Alive
I/II:2	F	1	37	130	δ-SPD pattern	Decreased	Reduced number of δ-granules by EM	NP	AML-M2 45	8 years	Alive
I/II:3	F	0	38	140–160	δ-SPD pattern	Decreased	NP	NP	No hematological malignancy	-	Alive
I/III:2 proband	M	2	1	90–120	δ-SPD pattern	Decreased	NP	Dysmegakaryopoiesis	No hematological malignancy	-	Alive
I/III:3	F	1	3	160 Normal platelet count	δ-SPD pattern	Decreased	NP	Dysmegackryopoiesis	No hematological malignancy	-	Alive
Patient J	F	0	10	50–60	δ-SPD pattern	Decreased	Mepacrine and CD63 decreased	NP	No hematological malignancy	<1 year	Alive
PatientK	F	0	1	40–50	NP	NP	NP	NP	secondary AML to MDS 13	12	Death

#### Pedigree A

Thrombocytopenia was detected in A/II:2 during her first pregnancy at the age of 26. This thrombocytopenia was also present in her three year old daughter (A/III:2), her brother (A/II:1) and father (A/I:2) who first developed acute myeloid leukaemia (AML) and deceased of an unclassified AML(UC-AML) six years after HSCT at the age of 39.

#### Pedigree B

Thrombocytopenia had not been diagnosed in B/III:6 before the occurrence of AML-M0 at the age of 41. Thrombocytopenia was then evidenced in her father (B/II:2), brothers (B/III:2; B/III:4), sister (B/III:8) and nephew (B/IV:3). Five members were affected by AML: B/II:1 (UC-AML); B/III:2 (AML-M5); B/III:4 (AML-M5); B/III:6 (AML-M0); B/IV:2 (AML-M4).

#### Pedigree C

C/II:1 presented with thrombocytopenia when he was 25 years old. Sixteen years later, he was diagnosed with T- acute lymphoblastic leukaemia (T-ALL). He achieved complete remission but developed AML-M1 and passed away 2 years later. FPD/AML was suspected because his mother (C/I:1) presented with thrombocytopenia when she was 46 and eventually died of AML secondary to MDS; his brother (C/II:3) died of AML-M1 at the age of 15; so far, his sister (C/II:2) has only thrombocytopenia.

#### Pedigree D

Thrombocytopenia was detected at the age of 8 as D/III:8 presented with velum petechiae and bruises and later developed T-ALL at the age of 28. He successfully underwent HSCT using as a donor his unaffected sister (D/III:9) who was found to be *RUNX1* wild-type. FPD/AML was suspected since his father D/II:6 presented with AML-M4 and his grandfather D/I:2 presented with an UC-AML when he was 60. Of note, D/II:1 and D/II:3 also had the mutation but a normal platelet count.

#### Pedigree E

Thrombocytopenia associated with δ-granule release defect was diagnosed the same year in several members as shown in Table 1. E/II:2 presented at the age of 47 with AML with fatal outcome. E/II:3 presented at the age of 54 with extensive buccal neoplasia and worsening thrombocytopenia and succumbed. Bone marrow analysis had showed marked trilineage dysplasia with rare, hypolobulated megakaryocytes and strongly basophilic cytoplasm, leading to the diagnosis of MDS with trilineage dysplasia. This familial association of δ-granule release defect with haematological neoplasia led us to analyse the *RUNX1* gene.

#### Pedigree F

F/II:6 had a lifelong history of easy bruising with thrombocytopenia since childhood. She developed AML-M5 at the age of 55, underwent HSCT from her *RUNX1* non-mutated sister (F/II:4) and achieved persistent complete remission. Familial thrombocytopenia (F/III:1; F/III:2) and propensity to develop malignancies (F/III:2-T-ALL and AML-M0 at the age of 18 and 23 old years respectively; F/I:1-CLL) was suspected. Her daughter (F/III:2) underwent sister (F/III:1) (later found to be mutated for *RUNX1)* allogeneic HSCT with fatal outcome.

#### Pedigree G

Thrombocytopenia was incidentally discovered in G/II:1 at the age of 12 before surgery. His father (G/I:2) was known to be persistent thrombocytopenic.

#### Pedigree H

H/III:1 was first seen at the age of 2 because of purpura and found to be thrombocytopenic. No treatment was administered. Four years later, he returned to hospital because of purpura and pancytopenia: an AML-M2 was then diagnosed. He underwent successful HSCT from HLA identical unrelated donor and is still in remission 10 years later. His mother (H/II:2), who had neither bleeding nor physical abnormality, was found to be slightly thrombocytopenic.

#### Pedigree I

I/III:2 was first seen at the age of one year old because of purpura and bruises, leading to the detection of thrombocytopenia. Several members of the family were found to be thrombocytopenic: his mother I/II:2, maternal aunt I/II:3, grandfather I/I:2 but not his cousin I/III:3. His maternal uncle (I/II:5) had thrombocytopenia and mild bleeding signs during infancy. I**/**II:2 developed AML-M1 at the age of 45 and successfully underwent HSCT.

#### Patient J

This child was first seen at the age of 10 to explore speech and developmental delay, hypotonia, associated with short stature and microcephaly. Numerous morphological abnormalities were noticed. Her platelet count was low but she had no history of bleeding. Family history was unremarkable.

#### Patient K

The propositus presented with craniofacial dysmorphism including epicanthus, wide nasal root, low set ear, ventriculoseptal defect, for which surgical correction was performed without bleeding, and a severe psychomotor retardation. When she was one year old, thrombocytopenia was detected and considered to be immune. At the age of 13, she was hospitalised for pancytopenia leading to the diagnosis of refractory anaemia with blasts excess type 2. AML appeared seven months later. She successfully underwent sister allogeneic HSCT which was followed by AML relapse three years later. No other family members were found to be affected with thrombocytopenia or haematological malignancy.

### Platelet functional phenotypes

For the patients who developed leukaemia, data were obtained before its occurrence. MPV and platelet size were normal for all affected patients.

Platelet functional studies were carried out for 20 patients from eight unrelated pedigrees (A to I except B) and for patient J (Table 1). No such studies were performed for subjects belonging to pedigree B and for patient K, because samples were not available.

LTA was realized in the majority of cases. Second wave of aggregation in response to ADP was absent (except in pedigrees A and C), associated or not with a decreased aggregation in response to AA, epinephrine and collagen. These patterns suggested defective δ-granules release which was consistent with the findings for 10 patients evaluated with other assays (Table 1): decreased δ-granules by fluorescence or electron microscopy; reduced ATP release; reduced uptake and release of fluorescent mepacrine and decreased CD63 membrane expression after stimulation (Table 1). No defect was detected for the other explored glycoproteins (CD42b, CD41, CD62P).

### Megakaryocyte morphology

Morphological analysis of bone marrow smears showed marked dysmegakaryopoiesis in four families (C/I:2, C/II:1, C/II:2, E/II:3, H/III:1, I/III:2 and I/III:3) (Table 1). This dysmegakaryopoiesis consisted in the presence of hypolobulated megakaryocytes with high nucleo-cytoplasmic ratio, immature megakaryocytes with high nucleo-cytoplasmic ratio, strongly basophilic cytoplasm and poorly lobulated nuclei in association with micromegakaryocytes.

### *RUNX1* analysis

**Fig. 2:**
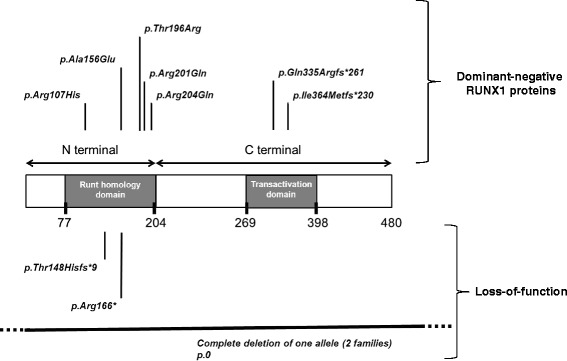
Position of the mutations detected in the *RUNX1* gene

Overall, 43 patients were found to be affected according to Sanger sequencing or CGH-array data (Fig. 1). The genetic alterations consisted of five missense mutations located to the runt homology domain (RHD), one nonsense and three frameshift mutations (Table 2). The position of these mutations in the *RUNX1* gene are shown in Fig. 2. There was no recurrent mutation, each one being unique to a given family. In addition, we found two complete deletions of the *RUNX1* locus (patients J and K). The predicted protein changes and their consequences (dominant negative or haploinsufficiency) are mentioned in Table 2. The effects of the *RUNX1* changes on pre-mRNA splicing and mRNA translation were not studied in vivo.

**Table 2:** Genotypic data and relationship with leukaemia

Pedigrees and patients	Number of affected patients/number of leukaemia cases and type of leukaemia (age at diagnosis)	Type of germline RUNX1 mutation and predicted change at protein level^(a)^	Localisation	Sequence change^(b)^		Predicted effect	Acquired RUNX1 Mutation at AML stage (Ref [11, 33])
				c.DNA	protein		
Pedigree A	4/2 AML-M0 and UC-AML (33–39 y)		exon 4	c.320G > A	p.Arg107His		
Pedigree B	8/5. UC-AML, AML-M0, AML-M4, 2 AML-M5 (6–42 y)		exon 5	c.467C > A	p.Ala156Glu		p.Arg129Ser Duplication of mutated allele
Pedigree C	4/4 secondary AML to MDS, 2 AML-M1, T-ALL (15–56 y)	- substitution - missense within the RHD	exon 6	c.602G > A	p.Arg201Gln	Dominant Negative RUNX1 protein	
Pedigree D	5/3 UC-AML, AML-M4, T-ALL (28–60 y)		exon 6	c.611G > A	p.Arg204Gln		p.Ala160Thr
Pedigree E	7/2 AML, MDS (47–53 y)		exon 6	c.587C > G	P.Thr196Arg		
Pedigree F	4/4 CLL, AML-M5, T-ALL and AML-M0 (18–55 y)	- insertion (frameshift) - normal RHD, severely truncated TAD	exon 9	c. 999_1003dup	p.Gln335Argfs261		p.Gly138ProfsX12
Pedigree G	2/0	- deletion (frameshift) - normal RHD,severely truncated TAD	exon 9	c. 1092del	p.Ile364Metfs230		
Pedigree H	2/1 AML-M2 (6 y)	- deletion (frameshift) - truncated RHD, no TAD and a small abnormal C terminal peptide	exon 5	c. 442_449del	p.Thr148Hisfs9		p.Thr121HisfsX9
Pedigree I	5/1 AML-M1 (45 y)	- substitution (nonsense) - premature termination: severely truncated protein, shorter RHD, no TAD	exon 5	c. 496C > T	p.Arg166X	Loss of function	p.Arg166X+ (LOH)
Patient J	1/0		3.4 Mb deletion in 21q22.12	NA	p.0		
Patient K	1/1 secondary AML to MDS (14 y)	- complete deletion of *RUNX1* - p.0 (haploinsufficiency)	2.16 Mb deletion in 21q22.12	NA	p.0		

Sequence variants were numbered starting from the first base of the ATG codon, numbering based on reference sequence NM_1754.4 containing 9 exons with 8 coding exons

^a^: The effect of the mutation at the protein level was not tested *in vitro* as we did not perform any functional analyses. This classification is based on the type and/or position of the mutations affecting the *RUNX1* coding region

RHD: runt homology domain; TAD: trans-activation domain; LOH: loss of heterozygosity

^b^: Theoretical effect at the protein level (the effects of the *RUNX1* mutation on pre-mRNA splicing and mRNA translation were not studied). Mutant premature stop codons often trigger nonsense-mediated mRNA decay. Nonsense and out-of-frame variations might correspond to null alleles

## Discussion

The addition of clinical and laboratory data from a large number of patients and families with FPD/AML is useful to gain further insight into this syndrome because: i) this platelet disorder is rare; ii) pedigrees reported up to now are small and affected members still alive are few. In this study we report on a national survey in France including two syndromic patients and nine pedigrees, of which three have not yet been published at all. Furthermore, we provide a more complete description of the cases that had been partially reported in previous studies.

Our survey underlines that to date, FPD/AML remains first and foremost discovered after the occurrence of a haematological malignancy in seven pedigrees (A, B, C, D, E, G, H) (Fig. 1, Table 1). Haematological malignancies included eighteen AML, three MDS, and three T-ALL. Of note, two individuals first developed T-ALL followed by AML-M1 (C/II:1) and M0 (F/III:2) and one suspected patient (A/I:1) developed AML and died of relapse six years after HSCT. The lapse of time to FPD/AML diagnosis varied from several months to 55 years and most frequently corresponded to the time elapsed between the diagnosis of thrombocytopenia and the diagnosis of haematological malignancy. A syndromic thrombocytopenia associated with mental retardation and/or cardiac abnormalities was the second clinical phenotype in two patients. In these two patients (patients J, K) and as discussed by Van der Crabben et al. [19] the complete deletion of not only *RUNX1* but also several nearby genes are likely to contribute to the syndromic features. An isolated autosomal dominant (AD) inherited thrombocytopenia with a lifelong mild bleeding tendency was the third type of presentation in two pedigrees (F, I).

In this study we confirm that this AD thrombocytopenia was moderate (>50 x10^9^/L) sometimes near the lower limit of normal range [7, 8, 20, 21] and that patients with complete *RUNX1* deletion had a lower platelet count than those with *RUNX1* mutation (Table 1) [19, 22]. Spontaneous bleeding was most often absent or mild and ISTH bleeding scores were less than 4 but one patient. These scores are low and several reasons might explain that. (i) At the present time we cannot use a specific bleeding assessment for detection of platelet bleeding disorders: those used, in particular ISTH-BAT derived from the score validated for detection of von Willebrand disease, are not predictive of the presence of platelet defects [23]. (ii) Inherited disorders are commonly associated with a milder bleeding phenotype that comprises spontaneous cutaneous bleeding and abnormal bleeding after surgery, trauma and childbirth. All the patients we reported on and with low bleeding scores have not had surgery: thus so the score is probably underestimated. We can stress that mutated women have had bleeding neither during their pregnancies nor after delivery. (iii) Since thrombocytopenia is often moderate, sometimes close to the lower limit of the normal range, even with a δ-granule secretion defect, the bleeding tendency could be very mild. Interestingly, within a given family, some patients with the mutation may have thrombocytopenia and bleeding, whereas others do not (D/I:1 and D/II:3; I/III:3). Moreover, for some subjects, platelet count may be normal and/or thrombocytopenia can appear later (F/II:1; I/I:2 and I/II:3). Therefore, in the context of unexplained familial bleeding tendency and/or familial haematological neoplasia, the screening of mutated patient cannot rely only on the platelet count as already pointed out in FPD/AML [24] and in other inherited platelet disorders. Another consequence is that the screening for a related donor for HSCT must be performed only by the analysis of *RUNX1* which should not be restricted to members with thrombocytopenia. It was suggested that a sex-chromosome FPD/AML predisposition allele may account for a male predominance of affected individuals [25]. In our cohort of 41 mutated subjects, excluding those with sporadic large deletion, there were indeed 23 affected males. Thrombocytopenia is secondary to bone marrow dysmegakaryopoiesis. In the literature, bone marrow investigation is poorly documented before the occurrence of myelodysplasia and/or leukaemia. When reported, dysmegakaryopoiesis is particularly important [20, 21, 24, 26] and is in our experience a clue for early diagnosis [27, 28]. The megakaryocytic dysplasia can be considered as preleukaemic abnormalities and reflects the abnormal megakaryopoiesis secondary to the germline *RUNX1* mutation. Indeed, Runx 1 is a transcription factor playing a key role not only in megakaryocyte maturation and differentiation but also in both ploidization and proplatelet formation [12]. In one patient (D/III:8) the platelet life span was normal, consistent with a defective platelet production (data not shown).

One important aspect is the assessment of platelet functions and more specifically dense granules release. Although different approaches were used among the centres, a δ-granule deficiency or release defect might be the most prevalent abnormality (Table 1). This defect cannot be detected by LTA alone and it is essential to evaluate platelet δ-granule release in order to ensure accurate diagnosis. More specific assays have been developed as ATP measurement simultaneously with LTA, mepacrine uptake and release measurement by cytometry, EM. When two of those tests were used, δ-granule release defect was detected in 10 patients before leukaemia. As already noticed [24], some affected family members had no bleeding history and platelet function studies were not always extensively performed (pedigrees A, B, patient K); so the frequency of the defective phenotype might be underestimated. Moreover, we found that the δ-granule release defect can be present not only in patients with bleeding but also in patients without bleeding history within the same family (pedigree I) and also in a carrier with a normal platelet count (I/III:3). Platelet ultrastructural studies can be helpful in some cases [29]. In our study, EM has been utilized only once (pedigree I) and showed a moderate reduction of δ-granules. In summary, if the number of patients having a δ − granule release defect is too low to define the link between platelet functional phenotype and mutated patient with accuracy, it nevertheless suggests the value to systematically analyse δ-granule secretion for the *RUNX1* mutated patients. Our study reflects the difficulties to use a same methodology in the different centres and points to the need for expert recommendations to standardize the available tests and for studies aimed at evaluating new methodologies as recently suggested [30]. Other numerous platelet abnormalities have been reported in FPD/AML including impaired platelet aggregation, decreased platelet spreading, decreased activation of alphaIIb-beta3, reduced protein phosphorylation of myosin light chain [31], decreased production of 12- hydroxyeicosatetraenoic acid or one specific protein kinase C isoform (PKC-θ) [9]. It is known that Runx1 regulates the expression of genes that could be directly or indirectly involved in platelet function [9] and δ − granule defect could not be the only explanation for the detected platelet dysfunction.

A major point is the link between mutations and development of leukaemia (Table 2). Molecular investigations revealed 11 deleterious *RUNX1* alterations of which six were previously published (pedigrees B, C, F, H, I and patient J) [11–13]. In pedigrees A, D, E and J the missense and frameshift mutations are novel unreported mutations in FPD/AML.

All disease-causing mutations in *RUNX1* are private as no recurrence has been reported so far. *RUNX1* mutations are distributed throughout the gene and can be divided into two main categories (Fig. 2; Table 2). Complete deletions of *RUNX1*, splice-site mutations but also nonsense and frameshift mutations in the N-terminal region result in the absence of DNA-binding ability and transactivating potential. These mutants are very likely to be loss-of-function alleles leading to haploinsufficiency at the protein level (Table 2: pedigrees H, I; patients J and K). In contrast, missense mutations in the runt homology domain, frameshift and nonsense mutants in the C-terminal region, in-frame mutations may lead to Runx1 proteins with dominant-negative effects (Fig. 2; Table 2: pedigrees A, B, C, D, E, F, G).

Few data are available concerning genotype-phenotype correlations in FPD/AML families. Michaud et al. [32] proposed that mutations with dominant-negative effect are associated with higher propensity to develop leukaemia. We found indeed a higher incidence of MDS/AL in the seven pedigrees with dominant-negative mutations (19 MDS/LA among 34 mutated patients) in comparison with the two pedigrees and the two patients with mutations acting via loss of function (three MDS/LA in nine mutated patients) (Table 2). For six of the patients who developed AML in pedigree B: B/III.4; B/III:6, pedigree D: D/II:6, pedigree F: F/III:2 and III:6, pedigree H: H/III.1, pedigree I: I/II:2 a second alteration of *RUNX1* has been evidenced as previously reported (Table 2) [11, 33]. These alterations are a new mutation on the second non-mutated *RUNX1* allele, a loss of heterozygosity, a duplication of the mutated allele. By contrast in two of the patients (C/II:1; F/III:2) who developed T-ALL [33] these biallelic alterations of *RUNX1* were not present. The secondary genetic events that contribute to leukemic transformation remain largely unknown. Recently, Yoshimi et al*.* reported that somatic mutation in CDC25C is a recurrent event in the early phase of leukemic progression of FPD/AML, which induces premature mitosis and genetic instability in haematopoietic cells carrying germline *RUNX1* mutation [34]. Adequate medical follow-up of *RUNX1* mutated patients with only an autosomal thrombocytopenia remains a matter of debate.

The last point of discussion is the differential diagnosis. Two other inherited platelet disorders predisposing to leukaemia belong to the same sub group as FPD/AML *ie* the subgroup of AD thrombocytopenias with normal platelet volume and dysmegakaryopoiesis. These disorders are linked to mutations in the 5′UTR part of the gene coding for ankyrin repeat domain 26 (*ANKRD26*) (THC2, OMIM 188000) [35, 36] or *ETV6* gene [4]. We have recently described that in patients’ platelets with FPD/AML there is a persistence of the MYH10 protein (pedigree C patients C/II:1, C/II:2; pedigree I patients I/II:2, I/III:2; patient J) [37]. The interest of this detection was underscored by another group [7]. At the present time this detection can be performed only by western blotting, a method not widely used by all the laboratories but we think it constitutes a good screening to perform a more targeted genetic testing of the patients.

## Conclusion

In conclusion, our study reported the largest group of patients with germline *RUNX1* alteration on a nation-wide basis. Our data clearly emphasize some clinico-biological associations, especially the possible link between a defective release of δ-granules and FPD/AML. This hallmark is of great importance for the genetic screening of patients presenting with isolated AD inherited thrombocytopenia with normal platelet volume and without familial history of leukaemia or MDS. An improved awareness of this platelet disorder and an earlier diagnosis are warranted although such a diagnosis raises many questions about genetic counselling and appropriate therapeutic management.

## Abbreviations

CLL: chronic lymphocytic leukaemia
CR: complete remission
N: normal
NA: not applicable
ND: not determined
NP: not performed
